# Reimagining the Intensive Care Unit as a Complex Adaptive System: A Systems Thinking Perspective

**DOI:** 10.7759/cureus.115305

**Published:** 2026-08-27

**Authors:** Agnishikha Choudhury, Gitu Joshi, Saurabh Saigal, Abhijit P Pakhare, Ankur Joshi

**Affiliations:** 1 Community and Family Medicine, All India Institute of Medical Sciences, Bhopal, Bhopal, IND; 2 Anesthesiology and Critical Care, Sudha Hospital and Medical Research Centre, Kota, IND; 3 Anaesthesiology, All India Institute of Medical Sciences, Bhopal, Bhopal, IND

**Keywords:** complex adaptive systems, critical care units, healthcare systems, intensive care unit, system dynamics, systems thinking

## Abstract

Modern intensive care units (ICUs) are highly complex environments characterized by dynamic interactions among patients, healthcare workers, technologies, logistics systems, administrative processes, and resource constraints. Traditional reductionist approaches to critical care often focus on isolated clinical variables and individual interventions, but may inadequately explain many persistent ICU challenges such as overcrowding, burnout, delayed discharges, healthcare-associated infections, and inefficient resource utilization. Systems thinking provides an alternative framework for understanding these interconnected and adaptive processes. This narrative review explores the ICU through the lens of systems thinking and conceptualizes critical care delivery as a complex adaptive system. Key systems concepts, including feedback loops, nonlinearity, delays, emergence, adaptation, and leverage points, are discussed in relation to ICU functioning. The review examines how these concepts influence major critical care domains including ICU occupancy, infection control, workforce dynamics, patient flow, logistics, antimicrobial stewardship, and healthcare costs. The potential utility of systems-based tools such as causal loop diagrams, stock-flow modelling, and system dynamics approaches in ICU analysis and decision-making is also discussed. Particular emphasis is placed on the emergence of system-level failures despite competent individual clinical care, highlighting the importance of understanding interactions rather than isolated events. Viewing the ICU as a complex adaptive system may provide deeper insights into operational inefficiencies, patient outcomes, and organizational resilience. Systems-informed critical care strategies can improve ICU performance by targeting interventions at high-leverage points. Integrating systems thinking into critical care practice, research, and policy may help develop more adaptive, efficient, and sustainable ICU systems.

## Introduction and background

Intensive care units (ICUs) are among the most complex environments in modern healthcare systems. Critical care delivery involves continuous interactions among severely ill patients, healthcare professionals, technologies, and healthcare systems. ICU outcomes are often shaped not only by individual clinical decisions but also by the broader behaviour of the healthcare system in which these decisions occur [1,2].

Traditional approaches to critical care analysis have largely followed reductionist models, in which complex clinical situations are broken down into isolated components, such as organ dysfunction, laboratory parameters, or disease-specific interventions. Although reductionism has contributed significantly to advances in critical care medicine, many persistent ICU challenges remain difficult to explain through isolated variables alone. Systems thinking offers an alternate and parallel conceptual framework for understanding such interactions [3,4]. From this perspective, problems such as ICU overcrowding, prolonged length of stay, healthcare-associated infections (HAIs), and burnout frequently emerge from interactions among multiple system components [1,5]. These interactions view the ICU as a complex adaptive system, and that helps to explain how people and other elements within the ICU continually respond to one another and adjust to changing conditions [3,6,7]. 

While other traditions are especially useful for understanding how work is carried out in practice, systems thinking draws attention to how different parts of the ICU are connected and how changes in one area may influence others. For instance, systems safety and resilience engineering examine how safety, failure, and adaptation emerge within complex work systems [8-10]. Another concept known as the human factors approach focuses on how people interact with their tasks, technologies, organisational structures, and working environments [11,12]. These perspectives overlap, but each brings a distinct emphasis. Systems thinking, on the other hand, offers the broadest lens by interconnecting and detecting the ability to shape one tradition to another tradition. The requirement to think from the systems perspective may be further understood through different scenarios fairly common in ICU settings. As patient numbers rise, staff workload also increases, placing greater pressure on routine care, including infection-prevention practices. If HAIs increase, patients may require longer ICU stays, which can further contribute to high occupancy and workload. An infection-control problem that initially appears isolated may therefore be linked to a wider set of factors involving patient flow, staffing, workload, infection risk, and length of stay [13,14].

With this background, this narrative review explores the ICU through the lens of systems thinking and argues that critical care delivery should be conceptualized as a complex adaptive system. The review mainly focuses on how adult ICUs function from an operational and organisational perspective, drawing on relevant literature from critical care and health systems. It does not specifically address paediatric or neonatal critical care, disease-specific clinical management, end-of-life care, family involvement, or ICU telemedicine. The review is aimed at clinicians, ICU administrators, health-system researchers, quality-improvement professionals, and policymakers who are interested in understanding ICU care as an interconnected system and identifying ways to improve how it functions. The review begins by setting out its scope and search approach. It then considers several everyday challenges in critical care. The later sections show how systems tools can help to diagnose these interconnected problems, including why changes may sometimes have unexpected effects. Finally, the review highlights areas where change may have the greatest impact and discusses the limitations of the current evidence and areas that need further study. The four domains examined in this review (length of stay, infection control, medicine and logistics, and cost of care) were identified through keyword co-occurrence network analysis of the ICU literature. The analysis showed that these domains were among the most closely interconnected themes in published research on ICU systems. 

Methods

This paper is a narrative review. The reporting follows the Scale for the Assessment of Narrative Review Articles (SANRA) criteria for narrative review quality [15]. The literature search was conducted using a Rapid Automated Literature Search (RALS) framework. RALS is a four-stage, semi-automated approach designed to reduce reliance on predefined search terms alone. In the first stage, initial keywords relevant to each ICU domain were used to search PubMed through the Easypubmed package in R (R Foundation for Statistical Computing, Vienna, Austria). In the second stage, keyword co-occurrence networks were generated from the retrieved titles and abstracts using Litsearchr. This identified additional terms that were strongly represented in the literature and helped expand the search strategy. In the third stage, PubMed was re-searched using the expanded keyword set. In the fourth stage, the conceptual relevance of the retrieved articles to each domain was assessed using a BERT (Bidirectional Encoder Representations from Transformers)-based natural language processing model. The search process was applied across three domains: length of stay (37 articles included from 77 retrieved), infection control (40 articles; 2019-2025), and medicine and logistics (26 articles from 89 retrieved). Articles were included when they addressed ICU-specific processes or outcomes and provided conceptual or empirical information relevant to systems thinking. Systematic reviews, observational studies, and foundational systems science literature were considered. No language restriction was applied. No formal study-level quality appraisal checklist was applied, which is consistent with the purpose of narrative rather than systematic synthesis. (Details of the search terms, filters, and domain-specific yields are provided in the Appendices.)

Scope of the review

The systems lens was used to organize interdependent ICU problems rather than to treat each domain as an isolated event. The principal applied domains are length of stay and patient flow, infection control and antimicrobial resistance, workforce dynamics, medicine and logistics, and cost/resource utilization. These domains were selected because they form recurrent and interconnected clusters within the literature. They also illustrate feedback, delays, accumulation, adaptation, and leverage points across clinical and operational processes. Topics such as ICU telemedicine, family involvement, end-of-life care, and disease-specific management are important but lie outside the operational scope of this review.

## Review

Systems thinking in critical care

In healthcare, systems thinking offers a framework for understanding how interconnected components dynamically interact to produce outcomes that cannot be adequately explained by analyzing individual elements alone [1,2]. In the ICU, patients, healthcare professionals, technologies, administrative processes, and biological processes continuously influence one another. The resulting behavior can be nonlinear, adaptive, and emergent. Systems thinking therefore shifts the focus from isolated parts toward the relationships that connect them.

The Integrated Whole

A system must be understood as an integrated whole rather than as fragmented components. In the ICU, no clinical process exists in isolation. Staffing constraints can affect monitoring, procedural timing, communication, and infection prevention; diagnostic or discharge delays can propagate across the unit and contribute to occupancy strain and congestion [1,3,13]. The practical implication is that improvement efforts should consider how a change in one part of the system alters conditions in another part of the system.

Feedback Loops

**Figure 1:**
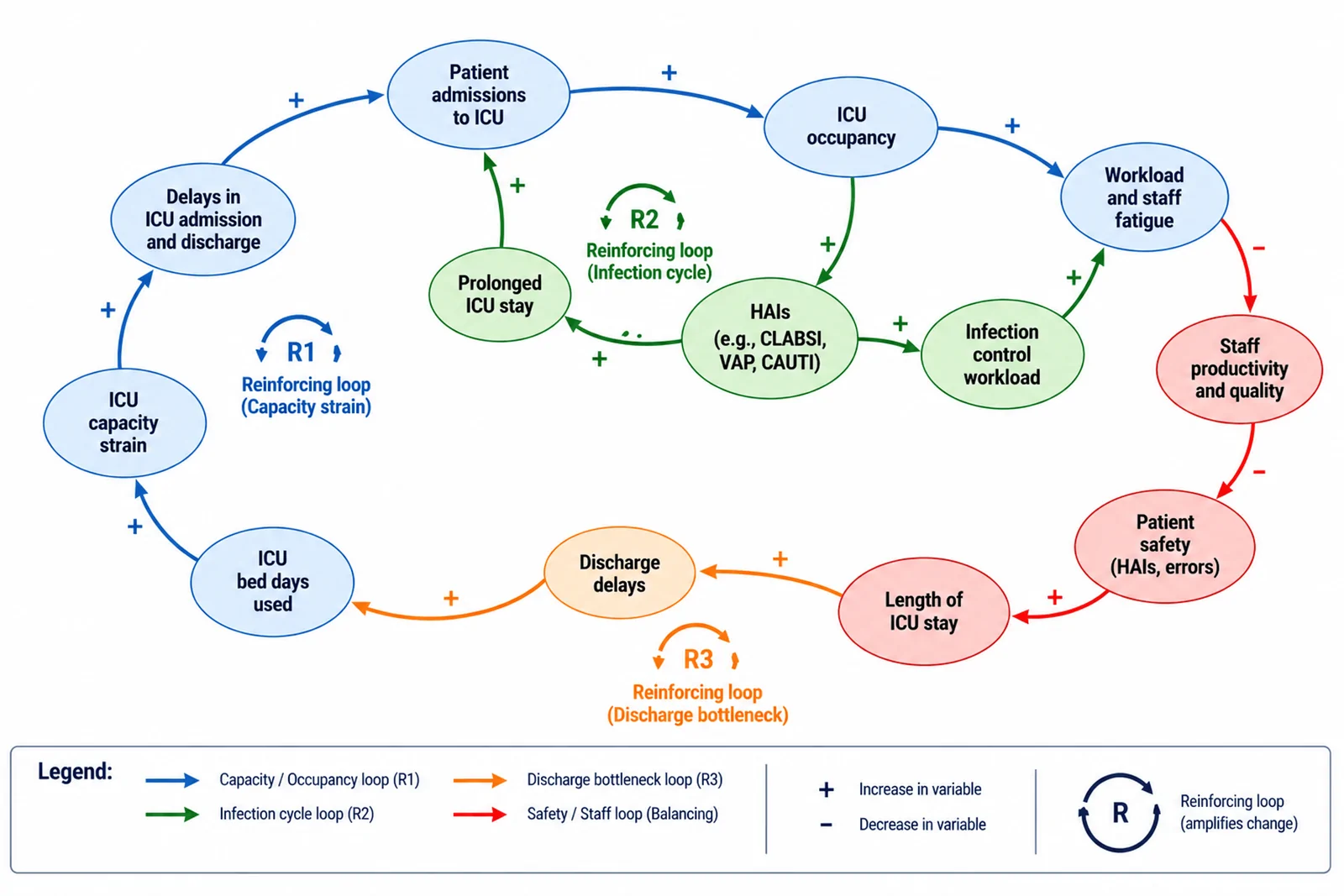
Causal loop diagram of ICU system dynamics HAI: healthcare-associated infection; CLABSI: central line-associated bloodstream infection; VAP: ventilator-associated pneumonia; CAUTI: catheter-associated urinary tract infection; ICU: intensive care unit Image Credit: Authors; created with R 4.6.1 binary for macOS 14 and R Studio as Interface  (R Foundation for Statistical Computing, Vienna, Austria)

Feedback loops are among the central organizing principles of systems behavior. Reinforcing loops amplify change, whereas balancing loops counteract change and promote stability [1,2,4]. A clinically relevant reinforcing loop can arise when increasing ICU occupancy elevates staff workload, reducing the capacity to maintain infection control. An increased burden of healthcare-associated infections can then prolong ICU stays, further increasing occupancy and workload [13,14]. Figure 1 illustrates this reinforcing structure, including the direction of relationships and delays through which its effects can accumulate over time.

Delays and Nonlinear Behaviour

Systems thinking also emphasizes delays between action and outcome. In ICUs, the consequences of operational changes are often not immediately visible. Delayed discharge, interruptions in antimicrobial optimization, staffing gaps, or procurement delays can produce downstream effects hours or days later. Nonlinearity means that the same disturbance can have different consequences depending on the state of the system. For example, a small staffing gap that is manageable at moderate occupancy can become destabilizing when demand approaches functional capacity [1,4,5,13].

Emergence and Adaptation

One defining characteristic of complex systems is emergence: the appearance of system-level behaviors that cannot be predicted solely by analyzing individual components. Adaptation describes how agents modify behavior in response to changing conditions. During resource strain, ICU teams reprioritize tasks, redistribute workload, modify workflows, and develop informal operational strategies to maintain functionality. These responses can stabilize care in the short term but can also create hidden vulnerabilities or long-term inefficiencies [3,6-8].

The ICU as a complex adaptive system

The ICU represents a vivid example of a complex adaptive system within healthcare [6,7,16]. Elements like admission demand, bed occupancy, workload, communication, procedural efficiency, infection prevention, patient outcomes, and length of stay are interdependent. Changes in one component can propagate through multiple pathways. This interconnectedness reflects what Perrow termed 'tight coupling' in high-risk systems [17]. This may be thought of as 'hidden coupling' to emphasise the less visible, indirect nature of dependency pathways within ICU networks.

Emergence and adaptation become clinically relevant in everyday ICU work. Team communication patterns, procedural efficiency, safety behaviors, and workarounds develop through repeated interactions. When patient load, staffing, equipment availability, or clinical priorities change, teams adapt by developing compensatory practices. Such adaptations can preserve immediate functionality, but can also introduce vulnerabilities that become embedded in routine practice.

The COVID-19 pandemic provided a large-scale real-world demonstration of the ICU as a complex adaptive system. Rapid surges in critically ill patients required hospitals to expand critical care beyond conventional ICU spaces, reorganize staff, redistribute supplies, and rapidly modify workflows. The experience also demonstrated that increasing physical bed capacity alone does not necessarily produce an equivalent increase in functional ICU capacity. Temporary staffing structures, task redistribution, altered patient-flow pathways, and other adaptive responses enabled ICUs to function under extraordinary demand. But it also introduced new operational dependencies and vulnerabilities. COVID-19 therefore illustrated how a major disturbance can propagate through interconnected components of critical care, requiring the system to continuously adapt in response [18-20].

In this review, the term ‘systems pathology’ is used descriptively to denote recurrent maladaptive patterns generated by dysfunctional interactions within the wider care system. Herein, it is not proposed as an established diagnostic category. A systems perspective shifts attention toward examination of the structures and conditions that repeatedly generate similar outcomes. Safety-I, which focuses on preventing things from going wrong, can be complemented by Safety-II, which examines how everyday adaptations happen inside the system [21]. Human-factors frameworks such as Systems Engineering Initiative for Patient Safety (SEIPS) similarly provide a structured way to examine interactions among people, tasks, tools and technologies, organization, and the physical environment [11,12].

Viewing the ICU as a complex adaptive system does not diminish the importance of individual clinical expertise. It places clinical decision-making within the operational environment in which care is delivered. Even highly skilled professionals work within systems that can enhance or constrain performance. Sustainable improvement in critical care therefore requires both high-quality clinical care and attention to the structures and interactions that shape its delivery.

The practical implications of these concepts can be translated into measurable features of routine ICU functioning. Table 1 summarizes key systems-thinking concepts, illustrative ICU examples, potential indicators that teams can monitor, and corresponding points for intervention.

**Table 1:** Translating key systems-thinking concepts into ICU practice. ICU: intensive care unit; LOS: length of stay; HAI: healthcare-associated infection

Systems Concept	Illustrative ICU Example	Potential Measurable Indicators	Potential Points for Action
Interdependence	High ICU occupancy increases workload and can affect communication, procedural efficiency, infection prevention, and patient flow.	Bed occupancy, staff-to-patient ratio, procedural delays, discharge delays	Coordinate staffing, bed management, procedures, and discharge planning rather than addressing each independently.
Feedback loops	Higher occupancy → greater staff workload → reduced infection-control adherence → increased HAI burden → longer LOS → further occupancy pressure.	Occupancy, workload, infection-prevention bundle adherence, device-associated infection rates, LOS	Interrupt the reinforcing cycle through workload management, infection-prevention measures, and timely discharge coordination.
Delays	A patient ready to leave the ICU remains because a ward or step-down bed is unavailable, maintaining ICU occupancy and limiting admission capacity.	Time from discharge readiness to transfer, delayed-discharge hours, ward/step-down availability	Begin discharge planning early, identify transfer bottlenecks, and strengthen real-time bed coordination.
Nonlinearity	A staffing deficit that is manageable at moderate occupancy can generate disproportionate strain when occupancy approaches functional capacity.	Occupancy trends, staffing ratios, overtime, missed/delayed care, adverse events	Establish escalation thresholds and activate surge staffing or workload redistribution before critical capacity is reached.
Emergence	Burnout, communication failures, and unsafe workarounds can arise from repeated interactions among workload, staffing, workflow, and organizational pressures.	Burnout measures, overtime, incident reports, communication failures; missed care	Examine recurring work-system patterns and contributing conditions rather than focusing exclusively on individual errors.
Adaptation	During strain, ICU teams redistribute tasks, modify staffing arrangements, and develop workarounds to maintain care delivery.	Task redistribution, staffing changes, overtime, protocol deviations, reported workarounds	Identify effective adaptations, formalize safe practices, and redesign potentially unsafe workarounds.
Stocks and flows	ICU occupancy is a stock determined by admission inflow and discharge, transfer, and mortality outflows.	Admissions/day, discharges/day, transfers/day, Occupancy, LOS	Manage inflows and outflows together and address downstream ward or step-down bottlenecks.
Leverage points	Changes in information flow, discharge decision-making, or organizational rules can influence several downstream processes simultaneously.	Discharge-decision time, transfer delays, communication failures, occupancy	Strengthen information flow, multidisciplinary coordination, escalation rules, and discharge pathways at structurally influential points.

Systems thinking applications in intensive care

The practical relevance of systems thinking becomes most apparent when examining recurring ICU problems as interconnected patterns rather than isolated events. The following domains illustrate how patient flow, workforce, infection risk, resources, and organizational constraints can interact over time.

ICU Occupancy and Length of Stay

**Figure 2:**
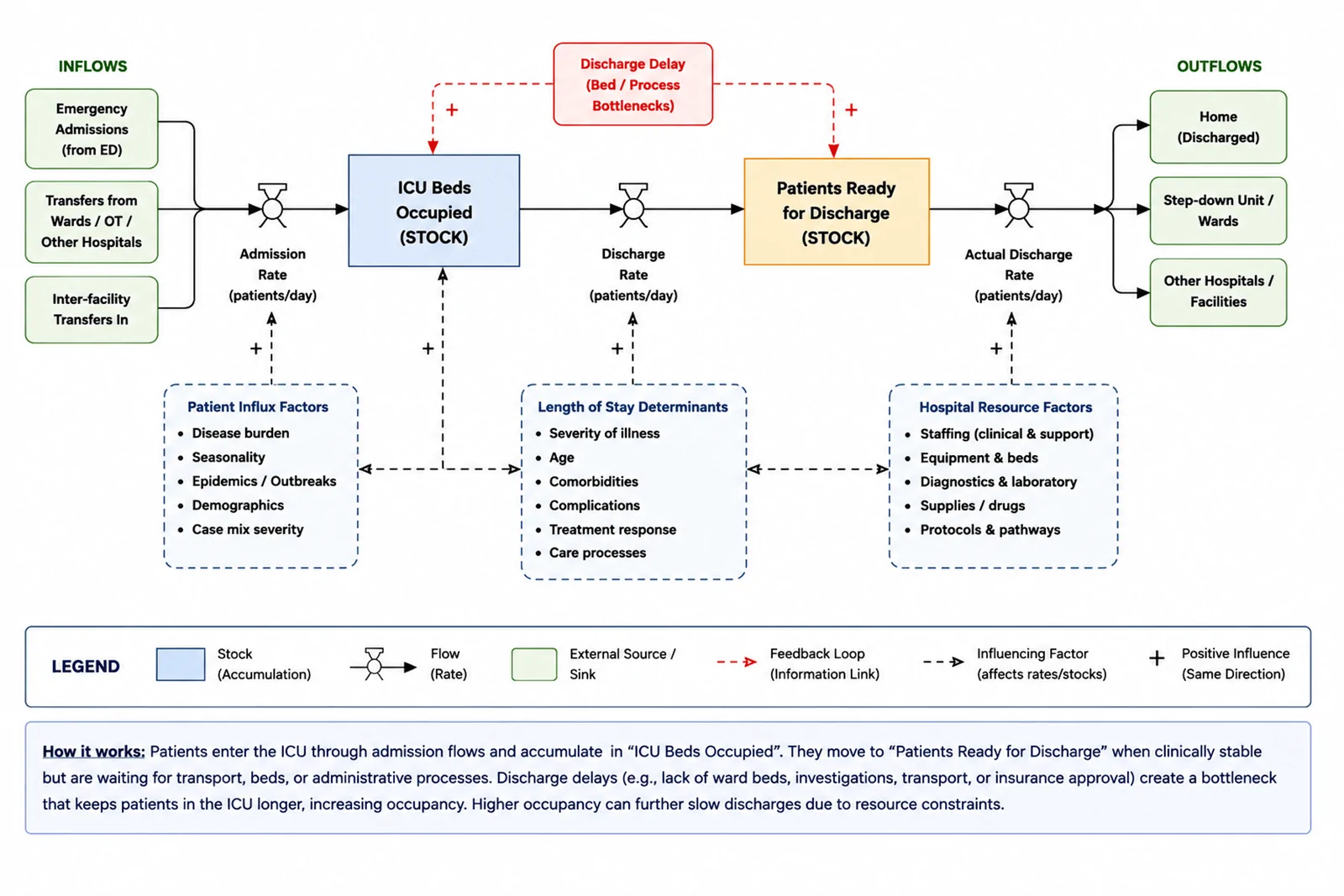
Stock-and-flow representation of ICU occupancy, admissions, outflows, and downstream transfer constraints Image Credit: Authors; created with R 4.6.1 binary for macOS 14 and RStudio as Interface  (R Foundation for Statistical Computing, Vienna, Austria)

ICU occupancy reflects the balance between patients entering and leaving the unit and can be conceptualized as a stock that changes through admission and discharge flows. Length of stay is therefore influenced not only by the patient's clinical condition but also by the efficiency of these flows. For example, a patient who no longer requires intensive organ support may nevertheless remain in the ICU while awaiting a ward or step-down bed. The delay keeps an ICU bed occupied and reduces capacity for incoming critically ill patients, contributing to congestion elsewhere in the hospital. Repeated across multiple patients, relatively small transfer delays can accumulate into sustained occupancy pressure [22,23]. Figure 2 represents this stock-and-flow structure and the downstream bottleneck.

HAIs and Antimicrobial Resistance

HAIs illustrate how infection risk is shaped by the wider ICU work system rather than by isolated infection-control behaviors alone. Occupancy strain, staffing levels, procedural intensity, cognitive workload, device utilization, antimicrobial exposure, and environmental conditions can interact to influence infection prevention and transmission. The reinforcing relationship between occupancy, workload, infection-control adherence, infection burden, and length of stay is summarized in Figure 1 [13,14,24].

Antimicrobial resistance similarly reflects interactions extending beyond individual prescribing decisions. Antimicrobial use is influenced by clinical uncertainty, diagnostic turnaround times, local resistance patterns, institutional prescribing practices, and the availability and responsiveness of stewardship systems. A systems perspective therefore places infection prevention and antimicrobial stewardship within the operational conditions in which clinical decisions are made [25,26].

Worked Example: A Central Line-Associated Bloodstream Infection (CLABSI) Cluster During Occupancy Strain

There is an ICU that experiences a sustained rise in admissions over several weeks while downstream ward capacity remains constrained. Occupancy rises, and delayed transfers accumulate. Staff workload increases, central-line device days increase, and routine infection-prevention work competes with urgent clinical tasks. A cluster of CLABSIs becomes apparent only after a delay because infection develops after exposure and surveillance indicators lag behind the operational conditions that produced the risk.

A conventional response might focus on the individual infection events or reinforce a single checklist. A systems analysis asks a broader set of questions: Which feedback loops are amplifying the problem? Which delays conceal the relationship between workload and infection? Which stocks are accumulating-occupied beds, device days, delayed transfers, or unfinished work? Which thresholds indicate that the unit is approaching functional overload? Figure 1 suggests a reinforcing loop in which occupancy pressure increases workload, reduced capacity for infection-prevention work increases infection burden, and infection prolongs length of stay, further increasing occupancy. Figure 2 shows how a downstream transfer bottleneck can maintain the stock of occupied ICU beds even when some patients are clinically ready to leave.

The example also changes where intervention is directed. Immediate infection-control reinforcement remains necessary, but a systems response would simultaneously examine staffing during occupancy peaks, device days, diagnostic turnaround, and downstream bed availability. Tracking these indicators together over time allows the team to test whether the proposed loop is supported by local data or not.

ICU Workforce Dynamics and Burnout

Increased patient acuity and occupancy can elevate cognitive burden, communication load, procedural demand, and emotional exhaustion among healthcare professionals. Fatigue and workload can impair communication and safety practices, while resulting inefficiencies can further increase workload and stress [27,28]. Adaptive responses can preserve immediate function, but chronic reliance on them can normalize overload and erode workforce resilience. Burnout prevention should therefore include work-system design, staffing adequacy, scheduling, team processes, and organizational support rather than relying only on individual wellness strategies.

ICU Logistics and Resource Flow

Resource availability in the ICU can also be understood through stock-and-flow behavior. Consider a critical medicine: available inventory represents a stock, procurement and replenishment increase that stock, and clinical use reduces it. If consumption temporarily exceeds replenishment, the resulting shortage can alter prescribing or delay treatment. The same principle applies to other finite ICU resources, including equipment, consumables, and oxygen supplies [20,29].

Resource constraints interact with workforce and patient-flow pressures. A shortage of appropriately trained personnel, for example, can reduce monitoring capacity or delay procedures during periods of high demand. Systems analysis therefore examines not only whether a resource shortage occurred but also how procurement, consumption, staffing, and operational demand interacted to produce it [13,18].

ICU Economics and Resource Utilization

Critical care is among the most resource-intensive domains of healthcare. Costs reflect disease severity and technological requirements, but they are also shaped by length of stay, mechanical ventilation, diagnostic and procedural delays, and the efficiency with which scarce resources are used [22,30,31]. A systems perspective therefore asks which operational structures generate avoidable resource consumption and whether redesign of patient flow, communication, prevention, or discharge processes can improve clinical and economic performance together.

Operational aspects of systems thinking for ICU analysis

Tools

Causal loop diagrams (CLDs) visualize reinforcing and balancing feedback structures by representing the direction and polarity of relationships among variables [1,2,5,32]. Figure 1 demonstrates this approach using ICU occupancy, workload, infection-control adherence, healthcare-associated infections, and length of stay. CLDs are primarily tools for representing hypotheses about system structure and for making proposed relationships explicit. A directional link should not, by itself, be interpreted as evidence of causation. Where empirical ICU evidence exists, proposed relationships should be evaluated against observed data. But where evidence is limited, the links should be regarded as testable hypotheses rather than established causal effects.

Stock-and-flow models complement CLDs by representing accumulation and movement within a system [2,4]. ICU occupancy can be conceptualized as a stock that changes through admission inflows and discharge, transfer, and mortality outflows. When downstream ward or step-down capacity constrains these outflows, patients accumulate within the ICU despite being clinically ready for transfer [22,23]. Figure 2 illustrates this structure and the resulting transfer bottleneck.

Unintended Consequences and System Adaptation

Interventions directed at one part of an ICU system can produce consequences elsewhere. Increasing admission capacity without corresponding staffing support, for example, can improve immediate access while increasing workload and reducing operational flexibility [13,18]. Short-term workarounds can likewise preserve immediate functionality while concealing structural problems that require longer-term redesign [8,21]. A systems perspective therefore considers both the intended effect of an intervention and the downstream responses it generates across the wider work system.

Leverage Points in Critical Care

Systems thinking shifts ICU improvement from repeated correction of downstream symptoms toward identification of the structures that generate recurring problems. Meadows' hierarchy of leverage points is useful because not all interventions have equal potential to change system behavior [1]. Changes to numbers or physical stocks such as adding a small number of beds or increasing a staffing target can be necessary but often sit at the lower end of leverage. Changes to delays, feedback strength, information flows, rules, goals, and underlying mental models can alter the way the system behaves more fundamentally.

Applied to ICU patient flow, adding beds can temporarily increase physical capacity, but the effect is limited if discharge delays, staffing constraints, or downstream bottlenecks remain unchanged. Improving real-time information about discharge readiness and downstream capacity acts at the level of information flow. Similarly, changing who is authorized to initiate or escalate discharge decisions changes system rules. Again, redefining the goal from maximizing individual bed occupancy to maintaining safe flow changes the objective guiding local decisions. At the highest level, shifting the mental model to 'patient flow is a hospital-wide system property' can redistribute responsibility and create broader solutions.

Communication, discharge coordination, workforce resilience, infection prevention, and antimicrobial stewardship therefore represent leverage areas. But the intervention chosen within each area should be judged by where it acts in the system. Structured multidisciplinary rounds and real-time information sharing can strengthen information flow. Anticipatory discharge pathways can reduce delays [22,23]. Staffing escalation rules can strengthen balancing feedback [13]; and stewardship systems can change both information and decision rules [25,26]. This ranking helps distinguish additive fixes from structural changes.

Practical Starting Points for ICU Teams

A practical systems-thinking exercise can begin with one recurring problem already recognized by the ICU team. The multidisciplinary team can map the problem together using a simple CLD, identify the main reinforcing and balancing loops, and mark significant delays. The purpose of Figure 1 is to make assumptions visible and create a shared representation of how the team believes the problem is generated.

The team should then select a small set of indicators that represent different parts of the proposed structure, for example, occupancy variation, delayed-discharge hours, device days, infection rates, boarding time, or staffing-ratio gaps, and examine them together over time. Repeated measurement allows the team to revise the map, test proposed relationships, and identify leverage points for interventions. SEIPS can support structured examination of the ICU work system and workflow [11,12], while STAMP (Systems-Theoretic Accident Model and Processes)/CAST (Causal Analysis based on Systems Theory) can provide a systems-oriented root-cause analysis after complex incidents [9,33].

Limitations

Several limitations of this review should be considered. First, although the literature search was structured to identify relevant evidence across the principal domains discussed, this is a narrative rather than a systematic review. The literature-selection process may therefore have introduced selection bias, and relevant studies or alternative systems perspectives may not have been captured. The synthesis should consequently be interpreted as a conceptually informed overview rather than an exhaustive assessment of the critical care literature.

Second, several relationships represented through systems concepts and causal loop structures in this review are conceptual rather than empirically established causal relationships. CLDs are useful for organizing complex interactions and generating hypotheses, but the presence and direction of an arrow do not demonstrate causation. The proposed feedback structures therefore require empirical testing using longitudinal and context-specific ICU data.

Third, systems models can be difficult to construct and validate using routinely collected ICU data. Clinical databases are generally designed around individual patients, treatments, and outcomes. This limits the ability to quantify some of the relationships proposed in systems models and highlights the need for data systems designed to capture operational processes as well.

Fourth, systems explanations carry a risk of post-hoc interpretation. Because adverse outcomes can often be linked retrospectively to multiple interacting factors, a systems explanation can become difficult to falsify unless relationships and expected system behaviors are specified in advance and subsequently tested. Systems models should therefore be treated as explicit, testable representations of hypothesized mechanisms.

Fifth, ICU structures, staffing models, resources, patient-flow pathways, organizational cultures, and surrounding health systems vary substantially across institutions and countries. Feedback structures and leverage points identified in one setting may therefore operate differently in another environment. Systems-informed interventions should be adapted to local context and evaluated before broader implementation.

Finally, emphasizing system-level contributors to adverse events should not eliminate individual professional accountability. Systems thinking helps us look beyond individual actions to understand the wider conditions that influence how people behave and how care is delivered. However, promoting safety in critical care also requires a balance between learning from system failures and recognising individual responsibility. A just-culture approach supports this balance by distinguishing unintentional human error or problems arising from system conditions from actions that are reckless or knowingly unsafe. This allows organizations to learn from mistakes and improve the system without removing accountability where it is appropriate [10,34].

Future Directions

As critical care becomes more data-intensive, technologically interconnected, and operationally complex, systems-informed approaches are likely to become increasingly relevant. Rising patient acuity, workforce shortages, antimicrobial resistance, financial constraints, aging populations, and public health emergencies create interacting pressures that are difficult to manage through isolated interventions alone.

Future critical care models can combine systems thinking with digital infrastructure, real-time analytics, simulation, and carefully designed predictive tools. Potential applications include occupancy forecasting, early warning of staffing strain, dynamic infection surveillance, and resource-allocation simulation [35,36]. These tools should support, rather than replace, clinical judgment.

The usefulness of predictive and simulation-based approaches depends on rigorous development, validation, and implementation. Models intended to support ICU decision-making should be evaluated for discrimination, calibration, and clinical utility. Wherever possible, they should be externally validated across different patient populations and healthcare settings. Their performance should also be monitored after implementation. Decision-support systems must additionally be integrated into clinical workflows in ways that minimize alarm fatigue. Predictive accuracy alone is therefore insufficient. Systems-based technologies should also demonstrate reliability and generalizability within the environments in which they are deployed [36].

Systems-informed research should also examine ICU survivorship, rehabilitation, and transitions of care as interconnected pathways extending beyond ICU discharge. Critically ill patients can experience prolonged physical, psychological, cognitive, and socioeconomic consequences after acute stabilization, and these outcomes are shaped by healthcare, rehabilitation, social, and economic systems [37].

Future work should move from descriptive conceptual models toward empirical testing of proposed feedback structures. Interdisciplinary collaboration among clinicians, systems scientists, engineers, administrators, epidemiologists, human-factors specialists, and public health professionals will be important for translating systems concepts into usable approaches.

## Conclusions

Many ICU outcomes arise not solely from individual clinical decisions but from interactions within the wider system in which critical care is delivered. Systems thinking provides a framework for understanding this complexity by shifting attention from isolated events toward relationships, feedback mechanisms, delays, adaptation, emergence, and patterns of behavior over time. Through this lens, persistent challenges such as overcrowding, prolonged length of stay, HAIs, workforce strain, operational congestion, and inefficient resource utilization can be understood as interconnected rather than independent problems.

This perspective complements rather than diminishes individual clinical expertise. High-quality critical care depends on skilled clinical decision-making; however, the effectiveness of those decisions is also shaped by staffing, workflows, information systems, resource availability, patient-flow pathways, and organizational structures. Recognizing these interactions creates opportunities to identify leverage points and address recurring structural drivers rather than repeatedly responding to downstream consequences. Integrating systems thinking into critical care practice, research, education, and policy provides a framework for developing more sustainable and patient-centered ICU systems. Future work should move beyond conceptual models toward empirical testing and evaluation of proposed feedback structures

## Appendices

Appendix A

**Table 2:** Overview of Search Parameters Across the Three Review Domains

Parameter	Domain 1: Length of Stay (LOS)	Domain 2: Infection Control (IC)	Domain 3: Medicine & Logistics (M&L)
Review type	Systematic-type (quantitative predictors)	Scoping review	Scoping review
Focus	Quantitative predictors and determinants of prolonged ICU length of stay in adult patients	HAI incidence and determinants; IPC practices; MDRO emergence; HCW knowledge and compliance with IPC protocols	Drug and consumable shortages; inventory management; wastage reduction; dispensing systems; supply chain resilience in critical care
Study designs	Cohort studies (prospective and retrospective observational)	Cross-sectional (47.5%), retrospective cohort (27.5%), prospective cohort, quality improvement studies, RCT	Quantitative or mixed-method designs with measurable supply chain outcomes
Database	PubMed	PubMed	PubMed
Search tool	Easypubmed R package (Stage 1)	Easypubmed R package + Litsearchr keyword network	Easypubmed R package + Litsearchr keyword network
Date filter	Last 5 years	2019–2025	Last 10 years
Access filter	Free full-text only	Free full-text only	Free full-text only
Initial yield (n)	77	Not separately reported	89
Abstracts screened (BERT)	57	Not separately reported	41
Studies included (final)	37	40	26
BERT model	google/bert-large-uncased-whole-word-masking-finetuned-squad (via reticulate R-Python bridge)	Same as LOS domain	Same as LOS domain
Language	English only	English only	English only

**Table 3:** Inclusion and Exclusion Criteria by Domain

Domain	Criterion	Inclusion criteria	Exclusion criteria
LOS	Inclusion / Exclusion	Adult ICU patients (≥18 years); cohort study design (prospective or retrospective); quantitatively reported predictors, risk factors, or determinants of prolonged ICU LOS; English language; open-access full text available	Non-ICU or general ward settings; case reports, editorials, letters, and narrative reviews without primary data; studies where LOS was a secondary or incidental outcome only; paediatric-only populations
IC	Inclusion / Exclusion	ICU or critical care unit setting; studies reporting on HAI incidence, risk factors, or IPC practices and outcomes; MDRO burden, antimicrobial stewardship, or HCW knowledge/compliance related to infection control; quantitative or mixed-method designs with measurable infection-related outcomes; English; published 2019 onwards	Non-ICU settings (community hospitals without critical care unit specification); studies focused solely on treatment efficacy without reference to IPC or prevention practices; qualitative-only studies and opinion pieces; studies published before 2019 or after 2025
M&L	Inclusion / Exclusion	ICU or critical care unit setting, or healthcare facility studies with explicit ICU-level supply data; studies addressing pharmaceutical or consumable supply chain management, inventory control, procurement, or dispensing systems; measurable outcomes (shortage rates, wastage volumes, dispensing errors, cost savings, or supply chain resilience indicators); English; last 10 years; open-access full text available	Studies focused solely on clinical drug efficacy or pharmacokinetics without any logistics or supply chain dimension; general hospital supply chain studies with no specific reference to critical care or ICU settings; qualitative-only studies and opinion pieces without quantifiable supply chain outcomes

**Table 4:** RALS Pipeline — Technical Overview of the Four Stages

Stage	Name	Tool / Method	Description
1	Keyword Search	Easypubmed R package; Boolean combinations of MeSH terms and free-text fields	Structured PubMed queries run via the Easypubmed R package. Boolean combinations of MeSH terms and free-text fields used to retrieve citations with abstracts. Domain-specific date and access filters applied at this stage.
2	Keyword Network & BERT Screening	Litsearchr (R) for co-occurrence network; BERT model: google/bert-large-uncased-whole-word-masking-finetuned-squad; called via reticulate R-Python bridge	Litsearchr used to build a keyword co-occurrence network from retrieved titles and abstracts. Terms with high centrality used to refine or confirm the search strategy. BERT model applied to screen abstracts for relevance against the study objective, replacing manual title-abstract screening.
3	Full-Text Retrieval	Programmatic web scraping; open-access PDFs only	Open-access full-text PDFs retrieved programmatically through web scraping for articles flagged by BERT screening. Articles behind paywalls or without an available manuscript were excluded at this stage.
4	Manual Full-Text Review	Manual review by primary reviewer; narrative synthesis by domain	Remaining articles assessed against full domain-specific inclusion and exclusion criteria. Data extraction carried out manually by the primary reviewer. Findings synthesised narratively by domain. No meta-analysis was performed.

Appendix B

https://github.com/ankurjoshi/review_additional_ref/blob/main/supplementary_references.md
